# Suppression of interferon β gene transcription by inhibitors of bromodomain and extra-terminal (BET) family members

**DOI:** 10.1042/BJ20141523

**Published:** 2015-06-15

**Authors:** Nazma Malik, Stefan Vollmer, Sambit Kumar Nanda, Marta Lopez-Pelaez, Alan Prescott, Nathanael Gray, Philip Cohen

**Affiliations:** * MRC Protein Phosphorylation and Ubiquitylation Unit, College of Life Sciences, Dow Street, University of Dundee, Dundee DD1 5EH, Scotland, U.K.; † Division of Cell Signalling and Immunology, College of Life Sciences, University of Dundee, Dundee DD1 5EH, Scotland, U.K.; ‡ Dana Farber Cancer Institute, Harvard Medical School, Boston, MA 02115, U.S.A.

**Keywords:** BI-2536, bromodomain and extra-terminal, histone, interferon, Polo-like kinase, Toll-like receptor

## Abstract

PLK (Polo-like kinase) inhibitors, such as BI-2536, have been reported to suppress *IFNB* (encoding IFNβ, interferon β) gene transcription induced by ligands that activate TLR3 (Toll-like receptor 3) and TLR4. In the present study, we found that BI-2536 is likely to exert this effect by preventing the interaction of the transcription factors IRF3 (interferon-regulatory factor 3) and c-Jun with the *IFNB* promoter, but without affecting the TBK1 {TANK [TRAF (tumour-necrosis-factor-receptor-associated factor)-associated nuclear factor κB activator]-binding kinase 1}-catalysed phosphorylation of IRF3 at Ser^396^, the dimerization and nuclear translocation of IRF3 or the phosphorylation of c-Jun and ATF2 (activating transcription factor 2). Although BI-2536 inhibits few other kinases tested, it interacts with BET (bromodomain and extra-terminal) family members and displaces them from acetylated lysine residues on histones. We found that BET inhibitors that do not inhibit PLKs phenocopied the effect of BI-2536 on *IFNB* gene transcription. Similarly, BET inhibitors blocked the interaction of IRF5 with the *IFNB* promoter and the secretion of IFNβ induced by TLR7 or TLR9 ligands in the human plasmacytoid dendritic cell line GEN2.2, but without affecting the nuclear translocation of IRF5. We found that the BET family member BRD4 (bromodomain-containing protein 4) was associated with the *IFNB* promoter and that this interaction was enhanced by TLR3- or TLR4-ligation and prevented by BI-2536 and other BET inhibitors. Our results establish that BET family members are essential for TLR-stimulated *IFNB* gene transcription by permitting transcription factors to interact with the *IFNB* promoter. They also show that the interaction of the *IFNB* promoter with BRD4 is regulated by TLR ligation and that BI-2536 is likely to suppress *IFNB* gene transcription by targeting BET family members.

## Abstract (precis)

We have found that interferon production is suppressed by compounds that prevent bromodomains from interacting with acetylated histones at the interferon gene promoter. This is a new way in which interferon production is regulated to combat bacterial or viral infection.

## INTRODUCTION

The interaction of viral dsRNA or bacterial LPS (lipopolysaccharide) with TLR3 (Toll-like receptor 3) or TLR4 respectively activates a signalling network that induces the production of IFNβ (interferon β, encoded by the *IFNB* gene). The activation of these receptors leads to the recruitment of the adaptor protein, TRIF [Toll/IL-1R (interleukin 1 receptor) domain-containing adaptor inducing IFNβ], which triggers the activation of TBK1 {TANK [TRAF (tumour-necrosis-factor-receptor-associated factor)-associated nuclear factor κB activator]-binding kinase 1} complexes by a mechanism that is not yet understood. Once activated, TBK1 complexes catalyse the phosphorylation of IRF3 (interferon-regulatory factor 3), which is followed by the dimerization of IRF3 and its translocation to the nucleus, where it binds to *IFNB* promoters to stimulate *IFNB* gene transcription [1–6]. The production of IFNβ by the TLR3–TRIF pathway is required for host defence against many viruses in mice, such as cytomegalovirus [7], and in humans is essential for protective immunity against HSV1 (herpes simplex virus 1) and HSE (HSV1 encephalitis). HSE, a rare and potentially fatal disease of the CNS (central nervous system), is caused by mutations in genes encoding components of the TLR3 signalling network, such as TRIF, TBK1, IRF3 and TLR3 itself [8–10].

The first traces of IFNβ formed by the TLR3 pathway bind to the Type1 interferon receptor (IFNAR), activating the JAK (Janus kinase) family members JAK1 and TYK2 (tyrosine kinase 2), which phosphorylate STAT1 (signal transducer and activator of transcription 1) and STAT2 [11]. These proteins form heterodimers that associate with IRF9 to form the ISGF3 (interferon-stimulated gene factor 3) complex, which binds to ISREs (interferon-stimulated response elements) in the promoters of ISGs (interferon-stimulated genes). This leads to increased expression of hundreds of proteins to mount an antiviral state within the cell. The ISGs include IRF7 [12], which can stimulate *IFNB* gene transcription either alone or as a heterodimer with IRF3 [13,14]. IRF7 also stimulates transcription of the genes encoding IFNα (interferon α), which can also activate the IFNAR. IRF7 therefore drives a positive-feedback loop that amplifies IFNβ production after prolonged exposure to viral dsRNA [14,15].

The PLKs (Polo-like kinases) have essential roles in cell division [16], and PLK1 is highly expressed in a variety of cancers [17–19], where it is associated with a poor prognosis. For this reason, specific PLK inhibitors have been developed that are undergoing clinical trials, such as BI-2536 [20], which does not inhibit several hundred other protein kinases that have been tested [21,22]. It was therefore surprising when BI-2536 and some other PLK inhibitors were reported to suppress the production of *Ifnb* mRNA and the transcription of some ISGs in primary BMDCs (bone-marrow-derived dendritic cells) stimulated with the dsRNA-mimetic poly(I:C) or LPS, or infected with VSV (vesicular stomatitis virus). Similar effects were observed in BMDCs from IFNAR-knockout mice, indicating that they occurred independently of the positive-feedback loop [23]. These intriguing observations led us to investigate how BI-2536 might be controlling IFNβ production. In the present paper, we report the results of these studies, which have revealed that this compound exerts its effects in a way that was not anticipated at the outset of this investigation.

## MATERIALS AND METHODS

### Materials

Poly(I:C) was purchased from Invivogen, LPS (*Escherichia coli* strain O5:B55) was from Alexis Biochemicals and IFNβ was from R&D Systems. BI-2536 was purchased from Axon. The BRD4 (bromodomain-containing protein 4) inhibitors JQ1, I-BET and I-BET151 were gifts from Dr James Bradner (Dana Farber Cancer Institute, Boston, MA, U.S.A.), whereas the TBK1 inhibitor MRT67307 was synthesized by Dr Natalia Shpiro (MRC Protein Phosphorylation and Ubiquitylation Unit, University of Dundee, Dundee, U.K.). The JNK1/2 (c-Jun N-terminal kinase 1/2) inhibitor JNK-IN-8 has been described previously [24]. The JAK inhibitor ruxolitinib was purchased from ChemieTek. The TLR7 agonist CL097 and the TLR9 agonist ODN1826 were purchased from Invivogen.

### Antibodies

Antibodies were raised in sheep against full-length BRD4 (sheep number S698D) and c-Jun (sheep number 702A) expressed in *E. coli* as GST-fusion proteins and the antiserum was affinity-purified against each antigen coupled covalently to agarose. The fourth bleed (sheep 698D) and second bleed (sheep 702A) were used for the studies reported in this paper. The anti-IRF5 antibody was raised in sheep (sheep S485D) as described in [25]. Antibody from the third bleed was used for all experiments. Phospho-specific antibodies recognizing IRF3 phosphorylated at Ser^396^ (catalogue number 4947), STAT1 phosphorylated at Tyr^701^ (catalogue number 9171), TBK1 phosphorylated at Ser^172^ (catalogue number 5483), ATF2 (activating transcription factor 2) phosphorylated at Thr^69^/Thr^71^ (catalogue number 9225), c-Jun phosphorylated at Ser^73^ (catalogue number 9164) and antibodies recognizing all forms of STAT1 (catalogue number 9172), TBK1 (catalogue number 3013), GAPDH (glyceraldehyde-3-phosphate dehydrogenase) (catalogue number 2118) and control IgG (catalogue number 2729) were from Cell Signaling Technologies. The phospho-specific antibody recognizing JNK1/2 phosphorylated at Thr^183^/Thr^185^ (catalogue number 44682G) and the antibody recognizing all forms of IRF3 (catalogue number 51-3200) were from Invitrogen. Alexa Fluor® 568-conjugated rabbit secondary antibody (catalogue number A10042) and Alexa Fluor® 488-conjugated sheep secondary antibody (catalogue number A11015) were from Life Technologies. Rabbit secondary antibodies conjugated to horseradish peroxidase were from Pierce (catalogue number 31460).

### Cell culture, cell lysis and immunoblotting

RAW264.7 macrophages (hereafter referred as RAW cells) and the human plasmacytoid dendritic cell line Gen2.2 (hereafter called Gen2.2 cells) were cultured as described in [25,26]. After stimulation with the ligands, the cells were washed with PBS and lysed with ice-cold lysis buffer [50 mM Tris/HCl (pH 7.5), 1 mM EGTA, 1 mM EDTA, 1% (v/v) Triton X-100, 1 mM sodium orthovanadate, 50 mM NaF, 5 mM sodium pyrophosphate, 0.27 M sucrose, 10 mM sodium 2-glycerophosphate, 0.2 mM PMSF and 1 mM benzamidine]. The lysates were centrifuged at 15000 ***g*** for 15 min at 4°C, and the supernatant, termed cell extract, was removed. Protein concentrations were determined using the Bradford assay. An aliquot of cell extract (20 μg of protein) was denatured in SDS, subjected to SDS/PAGE and immunoblotted as described in [27].

### Immunofluorescence microscopy

Immunofluorescence was carried out as described previously [28]. The cells were fixed in 4% (v/v) formaldehyde, permeabilized with 0.2% Triton X-100 in PBS (pH 7.4) and stained with an antibody recognizing all forms of IRF3 followed by Alexa Fluor® 568-conjugated secondary antibody. The cells were mounted using ProLong antifade reagent with DAPI (Molecular Probes, P-36931), and the images were collected on a laser-scanning confocal microscope (Zeiss LSM 700) with ten fields collected per coverslip. Images were quantified using the Volocity program (PerkinElmer). Nuclei were identified using the DAPI-stained channel while the mean intensity of IRF3 (red channel) in the nuclear region was measured. For each field, the mean nuclear intensity was calculated and used to calculate the overall mean nuclear intensity.

Gen2.2 cells were incubated for 1 h with inhibitors, and then stimulated for an additional 1 h with agonists. The cells were fixed for 10 min in 4% (v/v) formaldehyde and 50000 cells were centrifuged into pre-coated slides (Thermo Scientific). The cells were permeabilized by incubation with methanol for 10 min at −20°C, blocked for 1 h with 0.5% fish gelatin (Sigma–Aldrich) and 0.1% Tween 20 in PBS, then incubated for 16 h at 4°C with an anti-IRF5 antibody (2 μg/ml) [25] and washed with 0.1% Tween 20 in PBS at 21°C. After incubation for 1 h at 21°C with a secondary antibody (Alexa Fluor® 488-conjugated; 1:1000 dilution) and counterstaining with DAPI (0.05 μg/ml) to reveal nuclei, images were acquired using a Delta Vision DV3 deconvolution microscope with an oil-immersion ×63 objective lens and images were processed using OMERO. Images presented correspond to one stack from deconvolved three-dimensional images.

### Native polyacrylamide gel electrophoresis

Gels cast without SDS were pre-run for 30 min at 40 mA in 25 mM Tris/HCl and 192 mM glycine with and without 1% (w/v) sodium deoxycholate in the cathode and anode chamber respectively. Samples without SDS or a reducing agent were applied to the gel, electrophoresed for 60 min at 25 mA and transferred on to PVDF membranes. The membranes were blocked as described above for SDS/PAGE and immunoblotted using the antibody that recognizes all forms of IRF3.

### mRNA measurements

RNA was extracted from cells using the OMEGA Total RNA Kit, and 1.0 μg of RNA was reverse-transcribed using iScript reverse transcriptase and the accompanying reagents (Bio-Rad Laboratories), according to the manufacturer's instructions. qPCR (quantitative PCR) was then performed as described using the SsoFast™ EvaGreen® Supermix (Bio-Rad Laboratories). The primers used for measuring mRNA encoding mouse *Ifnb*, *Isg15* and *Cxcl10* [29] and *Il6* [30] have been described. The following primer pair was used for qPCR of the mouse *Rantes* (regulated upon activation, normal T-cell expressed and secreted) gene: Rantes-forward, 5′-GCTGCTTTGCCTACCTCTCC-3′ and Rantes-reverse, 5′-ACACTTGGCGGTTCCTTCG-3′. Normalization and quantification were performed using 18S RNA and the ΔΔ*C*_T_ method. All mRNA measurements were performed in triplicate.

### Measurement of IFNβ secretion

The level of secreted IFNβ in the cell culture medium was determined using the Verikine mouse and human IFNβ ELISA kits (PBL Interferon Source) or the LEGEND MAX™ Mouse IFN-β ELISA Kit (BioLegend) following the manufacturer's protocol.

### ChIP assay

RAW cells (1.5×10^7^) cells or Gen2.2 cells (1.6×10^7^ cells) were incubated for 1 h with inhibitors and stimulated with LPS or poly(I:C) (RAW cells) or CL097 (Gen2.2 cells) (see the Results section). The cells were then treated for 10 min at 20°C with 1% (w/v) formaldehyde and cross-linking was terminated by adding glycine to 0.125 M followed by washing the cells with PBS. The cells were lysed in 50 mM Tris/HCl (pH 8.1), 10 mM EDTA, 1 mM PMSF, Complete™ protease inhibitor cocktail (Roche) and 1% (w/v) SDS. Chromatin was sheared by eight 15 s bursts of sonication at 4°C using a VibraCell sonicator (Sonics) at 50% power (RAW cells) or by fifteen 30 s bursts at 4°C using a waterbath sonicator at high power (Bioruptor, Diagenode) (Gen2.2 cells). Samples were centrifuged at 15000 ***g*** for 10 min at 4°C, and the soluble chromatin fraction was diluted 10-fold in 20 mM Tris/HCl (pH 8.1), 2 mM EDTA, 150 mM NaCl and 1% (v/v) Triton X-100, and pre-cleared by incubation for 2 h at 4°C with Protein G–Sepharose and 2 μg of sheared salmon sperm DNA. After retaining 10% of the sample for use as an input control, the rest of the chromatin fraction was incubated for 16 h at 4°C with 5 μg of acetylated histone H3 (Millipore), 5 μg of anti-BRD4 antibody or 5 μg of anti-IRF3 antibody (Santa Cruz Biotechnology) or 2 μg of anti-IRF5 antibody or 5 μg of control IgG. To isolate the immune complexes, the samples were incubated for 1 h at 4°C on a rotating platform with 30 μl of Protein G-Sepharose. After brief centrifugation and washing once in 20 mM Tris/HCl (pH 8.0), 2 mM EDTA, 150 mM NaCl, 0.1% SDS and 1% (v/v) Triton X-100, once in the same buffer plus 0.5 M NaCl, once in 10 mM Tris/HCl (pH 8.0), 1 mM EDTA, 0.25 M LiCl, 1% (v/v) Nonidet P40 and 1% (w/v) sodium deoxycholate, and twice in 10 mM Tris/HCl (pH 8.0) and 1 mM EDTA, the immunoprecipitates were eluted with 0.1 M NaHCO_3_ and 1% (w/v) SDS, and cross-linking was reversed by incubation for 16 h at 65°C in 0.2 M NaCl. Samples were digested with Proteinase K (Qiagen) for 1 h at 45°C, and DNA was purified using a Spin Column PCR Purification Kit (NBS Bio). Purified immunoprecipitated DNA and input DNA were analysed by quantitative PCR using the SsoFast™ EvaGreen® Supermix. The primers for amplification of the mouse *Ifnb* promoter [29] and the human *IFNB* promoter [31] have been described. The qPCR data were analysed and presented using the Percentage Input {100×2[Input(*C*_T_)−IP(*C*_T_)]} method.

## RESULTS

### BI-2536 blocks *Ifnb* mRNA production without affecting the phosphorylation, dimerization or nuclear translocation of IRF3

**Figure 1:**
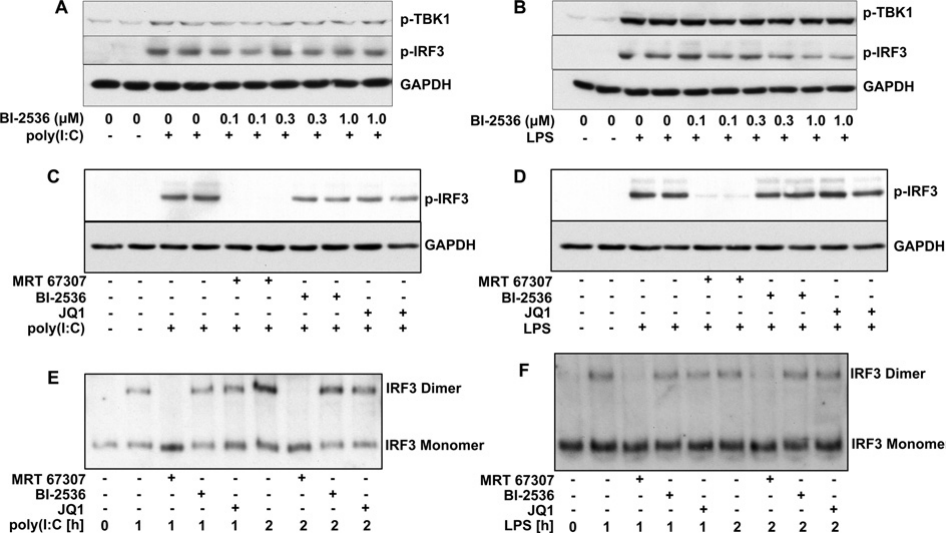
BI-2536 and JQ1 do not impair the poly(I:C)- or LPS -stimulated activation of TBK1 or the phosphorylation and dimerization of IRF3 (**A** and **B**) RAW cells were incubated for 1 h with the indicated concentrations of BI-2536, and then stimulated for 2 h without (−) or with (+) poly(I:C) (10 μg/ml) (**A**) or LPS (100 ng/ml) **(B)**. Cell lysates (25 μg of protein) were subjected to SDS/PAGE, transferred on to PVDF membranes and immunoblotted with antibodies that recognize TBK1 phosphorylated at Ser^172^, IRF3 phosphorylated at Ser^396^ and GAPDH as loading control. (**C** and **D**) RAW cells were incubated for 1 h without (−) or with (+) 2.0 μM of the TBK1 inhibitor MRT67307, 1.0 μM BI-2536 or 1.0 μM JQ1, and then stimulated for 1 h with poly(I:C) (10 μg/ml) (**C**) or LPS (100 ng/ml) (**D**). Cell extracts were immunoblotted with the anti-IRF3 and anti-GAPDH antibodies used in (**A**) and (**B**). Similar results were obtained in two other independent experiments for (**A**)–(**D**). (**E** and **F**) RAW cells were incubated for 1 h without (−) or with (+) 2.0 μM MRT67307, 1.0 μM BI-2536 or 1.0 μM JQ1 and then stimulated with poly(I:C) (10 μg/ml) (**E**) or LPS (100 ng/ml) (**F**) for the times indicated. The cell lysates (10 μg of protein) were subjected to native PAGE to separate the monomeric and dimeric forms of IRF3, which were detected by immunoblotting with an antibody that recognizes all forms of IRF3.

We confirmed earlier observations that BI-2536 prevents the LPS-stimulated secretion of IFNβ when included in the cell culture medium at a concentration of 1.0 μM or higher (Supplementary Figure S1A). Consistent with these observations, the production of *Ifnb* mRNA induced by either poly(I:C) (Supplementary Figure S1B) or LPS (Supplementary Figure S1C) was also prevented by the inclusion of BI-2536 (1.0 μM). In contrast, BI-2536 did not suppress the poly(I:C)-stimulated (Figure 1A) or LPS-stimulated (Figure 1B) activation of TBK1, as judged by the phosphorylation of its activation loop at Ser^172^ [32], or the phosphorylation (Figures 1A–1D), dimerization (Figures 1E and 1F) and nuclear translocation (Supplementary Figures S1D and S1E) of IRF3. In contrast, MRT67307, a potent and relatively specific inhibitor of TBK1 [33], blocked IRF3 phosphorylation (Figures 1C and 1D) and dimerization (Figures 1E and 1F) as expected. Our results disagree with the previous study in which BI-2536 was reported to prevent the nuclear translocation of IRF3 [23]. The compound JQ1, an inhibitor of the BET family of bromodomain inhibitors, which was included in these experiments for reasons discussed below, phenocopied the effects of BI-2536 (Figure 1).

The inclusion of BI-2536 in the cell culture medium had no effect on the IFNβ-stimulated phosphorylation of STAT1 at Tyr^701^ (Supplementary Figure S2A), indicating that it does not affect the interaction of IFNβ with IFNAR, or the activation or activity of JAK1 or TYK2. These findings indicated that the initial steps in the positive-feedback loop (see the Introduction) were unaffected by BI-2536.

We have reported that the poly(I:C)-stimulated production of *Ifnb* mRNA in primary bone-marrow-derived macrophages is unaffected up to 2 h by the potent and specific JAK inhibitors ruxolitinib and tofacitinib [29], whereas the late surge in *Ifnb* mRNA production that occurs after more prolonged stimulation with poly(I:C) is blocked by these compounds [29]. We also found that the LPS-stimulated production of *Ifnb* mRNA was essentially independent of the positive-feedback loop since it was unaffected by the JAK inhibitors at any time point [29]. These findings were confirmed in the RAW macrophage-like cell line in the present study. The poly(I:C)- or LPS-dependent increase in *Ifnb* mRNA production after 2 h was suppressed by BI-2536, but not affected significantly by ruxolitinib (Supplementary Figures S2B and S2C) at concentrations that completely blocked the JAK-catalysed phosphorylation of STAT1 at Tyr^701^ (Supplementary Figures S2D and S2E). Taken together, the results presented in Figure 1 and Supplementary Figures S1 and S2 indicate that BI2536 prevents poly(I:C)- or LPS-stimulated *Ifnb* mRNA production by a novel mechanism that is independent of either the classical TBK1–IRF3 signalling pathway or the JAK/TYK2–STAT1/2-driven positive-feedback loop.

### BI-2536 appears to exert its effects on *Ifnb* gene transcription by inhibiting BET family members

**Figure 2:**
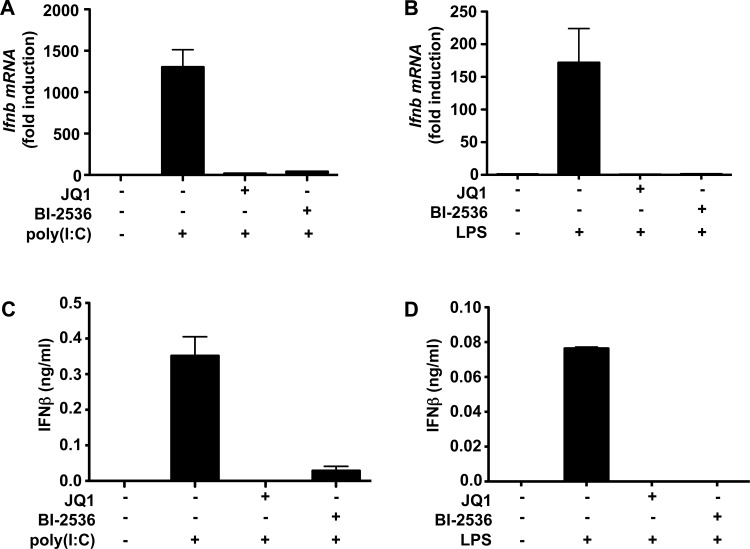
Poly(I:C)- and LPS-stimulated *Ifnb* gene transcription and IFNβ secretion is inhibited by BI-2536 and JQ1 RAW cells were incubated for 1 h without (−) or with (+) 1.0 μM JQ1 or 1.0 μM BI2536 and then stimulated for 8 h without (−) or with (+) poly(I:C) (10 μg/ml) (**A** and **C**) or for 4 h without (−) or with (+) LPS (100 ng/ml) (**B** and **D**). At each time point, the total RNA was extracted from the cells and *Ifnb* mRNA was quantified by qPCR (**A** and **B**) and the concentration of IFNβ in the cell culture medium was determined by ELISA (**C** and **D**). Results are means+S.E.M for triplicate determinations. (**A**) and (**B**) show the fold increase in mRNA levels relative to the values measured in cells that had not been stimulated with LPS or poly(I:C).

**Figure 3:**
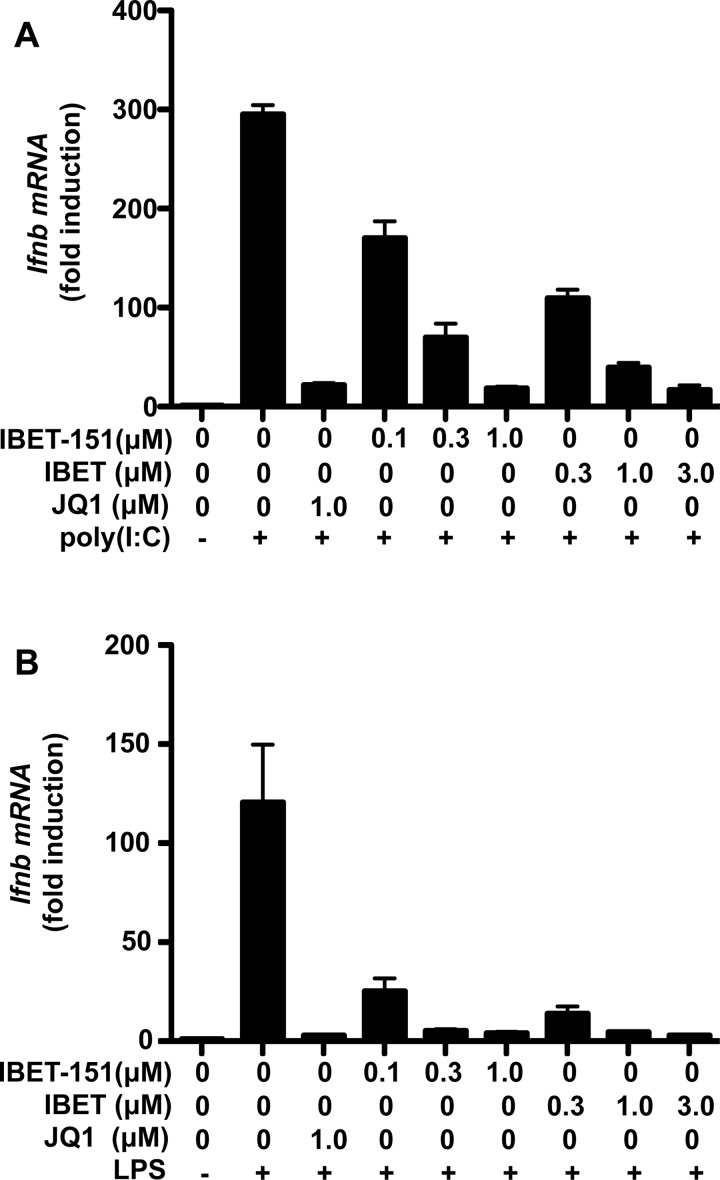
The BRD4 inhibitors I-BET and I-BET151 suppress poly(I:C)- or LPS-stimulated *Ifnb* gene transcription in RAW cells (**A**) Cells were incubated for 1 h with the indicated concentrations of I-BET151, I-BET or JQ1 and then stimulated for 2 h without (−) or with (+) poly(I:C). Total RNA was isolated and *IFNB* mRNA levels were quantified by qPCR. (**B**) As for (**A**) except that the cells were stimulated for 2 h with LPS (100 ng/ml). Results are mean±S.E.M. fold increases in mRNA levels relative to the values measured in cells not stimulated with poly(I:C) or LPS for triplicate determinations. Similar results were obtained in two independent experiments.

Although among protein kinases BI-2536 shows a high degree of specificity for PLK isoforms (see the Introduction), it was recently reported to bind strongly to BRD4 and other members of the BET family of proteins [34]. To investigate whether the effects of BI-2536 might be explained by the interaction of this compound with one or more BET family members, we initially compared its effects with those of JQ1, which is a potent inhibitor of the BET family [35]. We found that, whereas BI-2536 inhibited PLK1 *in vitro* with an IC_50_ value of 4.0±0.2 nM (average of duplicate determinations), JQ1 had no effect on PLK1 activity even at 10 μM, and at 1.0 μM did not inhibit 140 other protein kinases tested significantly (Supplementary Figure S3). Nevertheless, like BI-2536, JQ1 suppressed the poly(I:C)- or LPS-stimulated production of *Ifnb* mRNA (Figures 2A and 2B, and Supplementary Figures S2B and S2C) and IFNβ secretion (Figures 2C and 2D) as effectively as BI-2536, and without affecting the phosphorylation of IRF3 at Ser^396^ (Figures 1C and 1D), or the dimerization (Figures 1E and 1F) and nuclear translocation (Supplementary Figures S1D and S1E) of IRF3. Like BI-2536, JQ1 also suppressed the poly(I:C)- or LPS-stimulated phosphorylation of STAT1 at Tyr^701^ (Supplementary Figures S2D and S2E). Two other BET inhibitors, I-BET and I-BET151 [36], which are structurally unrelated to JQ1 or BI-2536, also prevented the poly(I:C)- or LPS-stimulated production of *Ifnb* mRNA (Figures 3A and 3B). Like JQ1, these compounds also had little effect on PLK1 activity with 10% inhibition (I-BET151) and 25% inhibition (I-BET) only at 1.0 μM *in vitro*.

### BET inhibitors impair the recruitment of IRF3, c-Jun and BRD4 to the *Ifnb* promoter

BET family members contain two bromodomains [35] and bind to pairs of acetylated lysine residues in the histone components of chromatin. BRD4 has been reported to recruit the pTEFb (positive transcription elongation factor B) kinase complex to transcription start sites, where it phosphorylates and activates RNA polymerase II to initiate transcription [37–40]. This can explain why some gene transcription programmes are inhibited when BET family members are displaced from chromatin by compounds that bind to their bromodomains.

**Figure 4:**
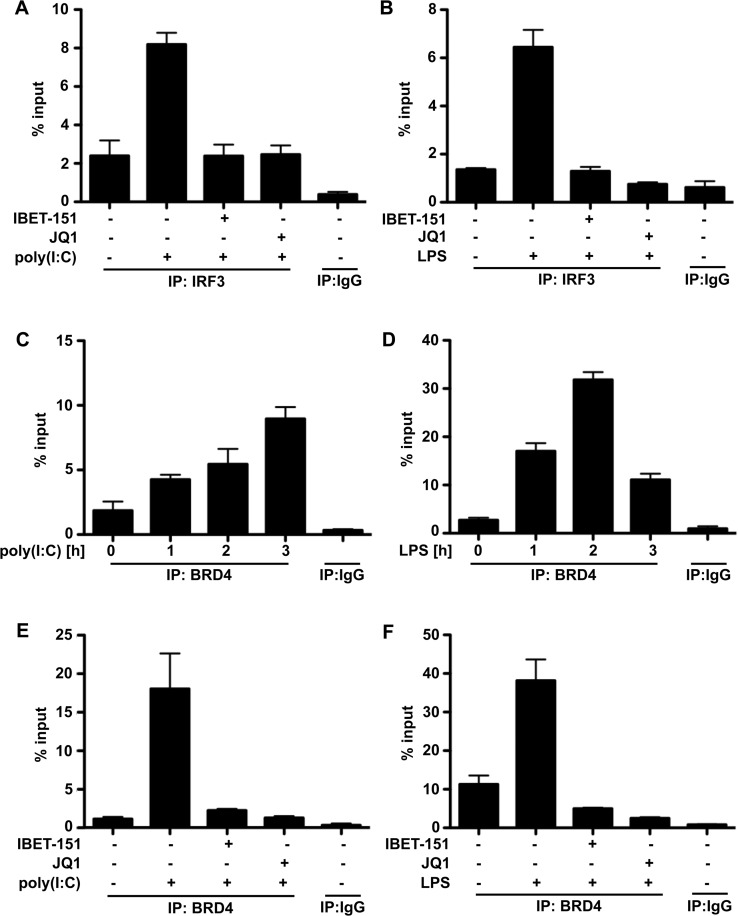
The poly(I:C)- or LPS-induced association of IRF3 and BRD4 with the *Ifnb* promoter is prevented by BET inhibitors RAW cells were incubated for 1 h, with or without JQ1 or I-BET151, then stimulated for the times indicated with either 10 μg/ml poly(I:C) (**A**, **C** and **E**) or 100 ng/ml LPS (**B**, **D** and **F**). They were then cross-linked and lysed, and the chromatin was sheared by sonication. ChIP was performed using antibodies that recognize IRF3 (**A** and **B**) or BRD4 (**C**–**F**). In all panels, the enrichment of the *Ifnb* promoter was measured by qPCR, normalized to input (see the Materials and methods section). Results are means+S.E.M. for triplicate determinations. IP, immunoprecipitation.

Interestingly, we found that the poly(I:C)- or LPS-stimulated association of IRF3 with the *Ifnb* promoter was suppressed by JQ1, I-BET151 (Figures 4A and 4B) and BI-2536 (Supplementary Figure S4A), suggesting that the interaction of one or more BET family members with acetylated histones permits IRF3 to access the *Ifnb* promoter. To investigate whether BRD4 was associated with the *Ifnb* gene promoter, we carried out further ChIP assays in which we immunoprecipitated BRD4 or control IgG and studied whether *Ifnb* gene promoter sequences could be detected in the immunoprecipitates. We found not only that these sequences were present in the immunoprecipitates, but also that the amount of *Ifnb* promoter DNA present in the immunoprecipitated BRD4 was increased by stimulation with poly(I:C) (Figure 4C) or LPS (Figure 4D). Importantly, this increase did not occur if the macrophages were incubated with the BET inhibitors I-BET151 or JQ1 (Figures 4E and 4F) or with BI2536 (Supplementary Figure S4B) before stimulation with poly(I:C) or LPS.

**Figure 5:**
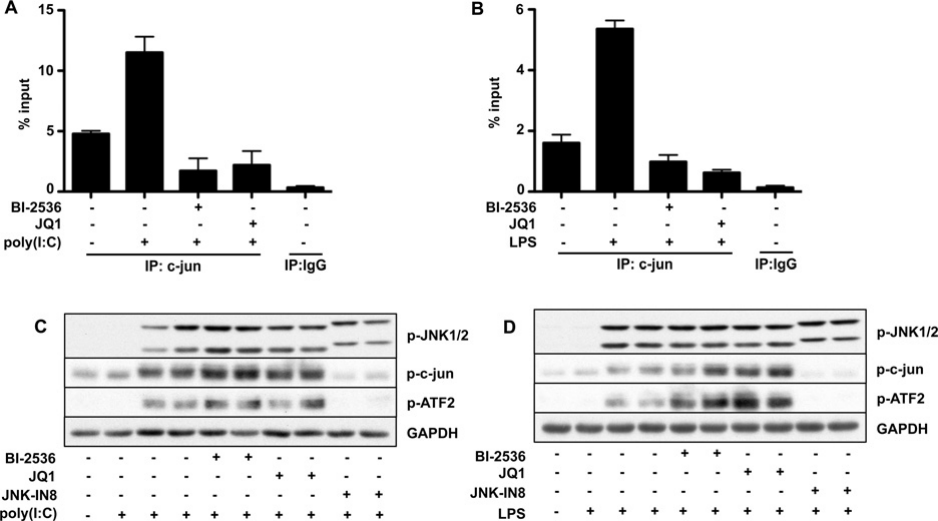
The poly(I:C)- or LPS-induced association of c-Jun with the *Ifnb* promoter is prevented by BET inhibitors without inhibiting the phosphorylation of c-Jun or ATF2 (**A** and **B**) The experiment was performed as in Figure 4 except that an anti-c-Jun antibody was used. (**C** and **D**) RAW cells were incubated for 1 h with 1.0 μM BI-2536, 1.0 μM JQ1 or 10 μM of the JNK inhibitor JNK-IN-8 and then stimulated for 1 h with 10 μg/ml poly(I:C) (**C**) or for 30 min with 100 ng/ml LPS (**D**). The cells were lysed and 20 μg of cell extract protein was denatured in SDS and subjected to SDS/PAGE followed by immunoblotting with antibodies that recognize the phosphorylated forms of JNK1/2, c-Jun and ATF2 as well as GAPDH. JNK-IN-8 binds covalently to JNK1 and JNK2 leading to a small decrease in their electrophoretic mobilities. IP, immunoprecipitation.

To check whether BET family members mediate IRF3 recruitment specifically or whether they have a more general effect on promoter accessibility, we also studied their role in recruiting c-Jun to the *Ifnb* promoter. c-Jun is a component of the AP1 (activator protein 1) transcription factor, which is reported to be a component of the IFNβ enhanceosome [41,42]. We found that the interaction of c-Jun with the *Ifnb* promoter was increased by stimulation with poly(I:C) or LPS and that basal, as well as stimulated, interaction with the *Ifnb* promoter was suppressed by either BI-2536 or JQ1 (Figures 5A and 5B). Therefore blocking the interaction of BET family members with acetylated proteins appears to have a more global effect on the accessibility of the *Ifnb* promoters to transcription factors.

Control experiments showed that neither BI-2536 nor JQ1 suppressed the poly(I:C)- or LPS-stimulated phosphorylation of JNK1/2 and that, in contrast with the covalent JNK inhibitor JNK-IN-8, they did not inhibit the phosphorylation of c-Jun and ATF2 (Figures 5C and 5D). Therefore BI-2536 and JQ1 do not suppress *Ifnb* gene transcription by inhibiting a component of the signalling pathway that leads to the activation of JNK and phosphorylation of its substrates.

### Influence of BI-2536 and JQ1 on the transcription of other poly(I:C)- and LPS-regulated genes

Since BI-2536 and JQ1 blocked IFNβ production by preventing the interaction of transcription factors with the *Ifnb* promoter, it was of interest to examine the effects of these compounds on the transcription of other poly(I:C)- and LPS-regulated genes. Similar to *Ifnb* gene transcription, we found that BI-2536 and JQ1 suppressed the transcription of *Il6* (Supplementary Figures S5A and S5B), suggesting that BET family members are also important in regulating the accessibility of transcription factors to the *Il6* promoter. However, the effects of these compounds on the transcription of *Rantes* (Supplementary Figure S5C and S5D) were modest, suggesting that other bromodomain-containing proteins may control the transcription of this gene.

### BET inhibitors block the TLR7- and TLR9-dependent production of IFNβ without affecting the nuclear translocation of IRF5

**Figure 6:**
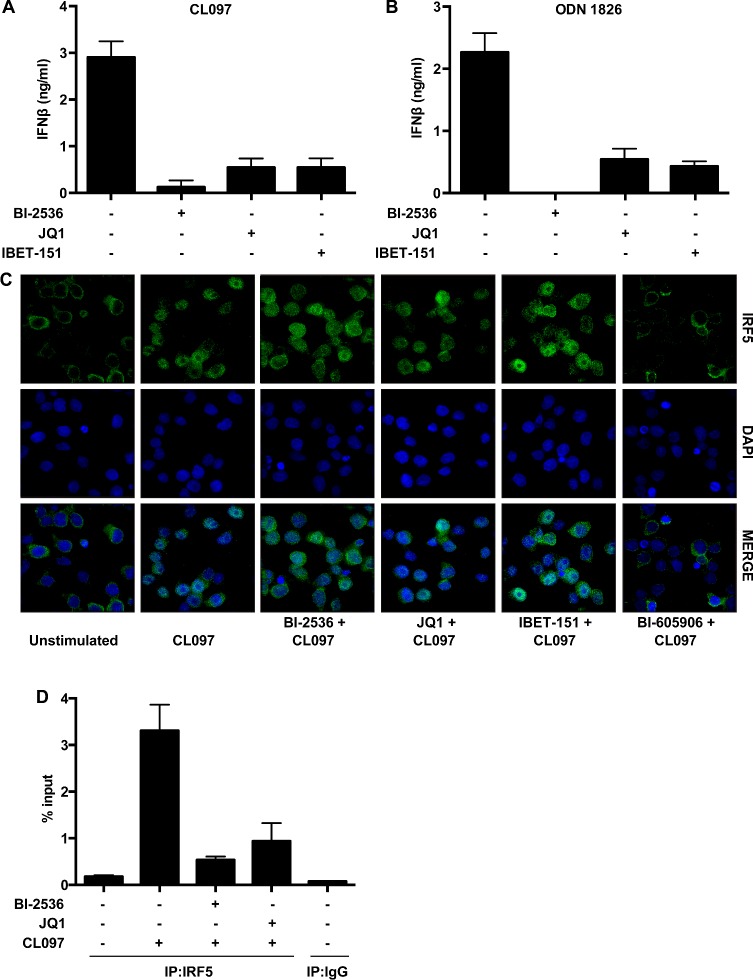
BET inhibitors block IFNβ production in Gen2.2 cells by suppressing the interaction of IRF5 with the *Ifnb* promoter and not by preventing the nuclear translocation of IRF5 (**A** and **B**) Gen2.2 cells were incubated for 1 h with or without BI-2536 (1.0 μM), JQ1 (1.0 μM) or I-BET151 (1.0 μM) and then stimulated for 8 h with CL097 (1.0 μg/ml) (**A**) or for 12 h with ODN1826 (1.0 μM) (**B**). The concentration of IFNβ in the culture medium was measured by ELISA. Results are means+S.D. from two independent experiments each performed in duplicate. (**C**) Gen2.2 cells were incubated for 1 h with or without BI-2536 (1.0 μM), JQ1 (1.0 μM), I-BET151 (1.0 μM) or BI-605906 (5.0 μM) and then stimulated for 1 h with CL097. Staining with anti-IRF5, or DAPI to reveal nuclei, followed by deconvolution microscopy was performed as described in the Materials and methods section. (**D**) Gen2.2 cells were incubated for 1 h with BI-2536 (1 μM) or JQ1 (1 μM), then stimulated for 1 h with the TLR7 agonist CL097 (1 μg/ml), cross-linked and lysed. Chromatin was sheared by sonication and ChIP was performed using anti-IRF5. The enrichment of the *Ifnb* promoter was measured by qPCR, normalizing to input. Results are means+S.D. similar results were obtained in three independent experiments each performed in duplicate. IP, immunoprecipitation.

We recently reported that the TLR7-stimulated production of IFNβ in the human plasmacytoid dendritic cell line Gen2.2 occurs via an analogous pathway in which IKKβ [IκB (inhibitor of nuclear factor κB) kinase β] phosphorylates IRF5 at Ser^462^, inducing its dimerization and translocation to the nucleus where it stimulates *Ifnb* gene transcription [25]. Similar to the TLR3–TBK1–IRF3 pathway, we found that BI-2536, JQ1 and I-BET151 blocked the TLR7- or TLR9-stimulated secretion of IFNβ in Gen2.2 cells (Figures 6A and 6B), without affecting the TLR-stimulated translocation of the endogenous IRF5 to the nucleus (Figure 6C). In contrast, BI-605906 blocked nuclear translocation of IRF5 as expected (Figure 6C). The antibody employed in these studies recognized IRF5 specifically because the signal was abolished by the siRNA knockdown of IRF5 (Supplementary Figure S6). We also found that stimulation with the TLR7 agonist CL097 induced the interaction of IRF5 with the *Ifnb* promoter and that this was blocked by either BI-2536 or JQ1 (Figure 6D).

## DISCUSSION

The work described in the present paper was prompted by a report that inhibitors of the PLK subfamily of protein kinases, such as BI-2536, prevented *Ifnb* gene transcription induced by LPS, poly(I:C) or viral infection [23], raising the question of how these kinases might control this process. We confirmed that BI-2536 suppressed *Ifnb* gene transcription induced by poly(I:C) or LPS, but found that it occurred independently of the classical TRIF-dependent pathway in which the activation of TBK1 is followed by the phosphorylation and dimerization of the transcription factor IRF3 (Figure 1 and Supplementary Figure S1). Moreover, and in contrast with the earlier report [23], we found that BI-2536 did not inhibit the translocation of IRF3 to the nucleus (Supplementary Figure S1D). We also established that BI-2536 was exerting its effect independently of the positive-feedback loop, which is driven by IFNβ via the JAK1/TYK2–STAT1/2 pathway (Supplementary Figure S2). On the other hand, BI-2536 did prevent the interaction of activated IRF3 (Figure 4) or the transcription factor c-Jun (Figure 5) with the *Ifnb* promoter, indicating that this compound was exerting its effect at the level of the *Ifnb* promoter distal to the activation of IRF3.

BI-2536 is a rather specific kinase inhibitor that does not affect several hundred other protein kinases that have been tested. However, after the experiments described in the preceding paragraph had been completed, we learned that a number of protein kinase inhibitors, including BI-2536, bind to the first bromodomain of the BET family member BRD4 and displace BRD4 and other BET family members from chromatin. These results, together with the three-dimensional structure of the BRD4–BI-2536 complex, have subsequently been published [34]. The two bromodomains of BET family members interact with pairs of acetylated lysine residues on histones in chromatin and are thought to facilitate specific gene transcription by recruiting other proteins to gene promoters. For example, BRD4 recruits the pTEFb kinase complex to transcription start sites, where it phosphorylates and activates RNA polymerase II to initiate transcriptional elongation [37–40]. These findings led us to study other compounds that interact with the bromodomains of BET family members, and which are structurally unrelated to BI-2536 or each other, and do not inhibit PLKs. We found that, similar to BI-2536, these compounds also suppressed TLR3-, TLR4-, TLR7- or TLR9-stimulated *Ifnb* gene transcription and secretion (Figures 2, 3 and 6) without affecting the activation or nuclear translocation of IRF3 (Figure 1 and Supplementary Figure S1D) or IRF5 (Figure 6). These results suggest that BI-2536 blocks *Ifnb* gene transcription by inhibiting BET family members and not the PLK subfamily of protein kinases. Consistent with this notion, we found that BI-2536 or BET inhibitors suppressed the interaction of IRF3 or c-Jun (Figures 4 and 5) or IRF5 (Figure 6) with the *Ifnb* promoter and that BRD4 was associated with the *Ifnb* promoter (Figures 4C and 4D). Thus BET family members have an important general role in permitting accessibility of transcription factors to the *Ifnb* promoter. BET family members appear to have a similar role in regulating the *Il6* promoter, but not the *Rantes* promoter (Supplementary Figure S5).

Interestingly, poly(I:C) and LPS enhanced the association of BRD4 with the *Ifnb* promoter and this was prevented by the bromodomain inhibitors (Figures 4C–4F). To our knowledge, this is the first report that the association of a BET family member with a gene promoter is regulated by ligands that activate TLRs. This increased association of BRD4 with the *Ifnb* promoter could be explained by an increase in histone acetylation, which in turn could arise from increased HAT (histone acetyltransferase) activity and/or decreased HDAC (histone deacetylase) activity. Importantly, the HAT activities of CBP [CREB (cAMP-response-element-binding protein)-binding protein] and PCAF (p300/CBP-associated factor) are reported to be needed for *Ifnb* gene transcription [43,44]. Whether TLR ligands enhance the interaction of BRD4 with the *Ifnb* promoter by activating these or other HATs and/or by inhibiting HDACs is unknown, but viral infection has been reported to induce the localized hyperacetylation of histones at the *Ifnb* promoter [45]. Alternatively, or in addition, LPS and poly(I:C) might induce a modification of BRD4 that enhances its ability to interact with acetylated histones, or these TLR agonists might stimulate the synthesis of BRD4 and other BET family members within cells.

The finding that compounds developed as protein kinase inhibitors interact strongly with the bromodomains of BET family members and prevent their interaction with acetylated lysine residues [34,46] has far-reaching implications. It implies that many protein kinase inhibitors reported to suppress gene transcription, or other events dependent on gene transcription, may actually exert these effects by inhibiting BET family members and not protein kinases. The human genome encodes 42 proteins that contain bromodomains (56 bromodomains in total) and the development of a panel of these bromodomains will clearly be essential to assess which protein kinase inhibitors possess these ‘off-target’ effects. The present study has shown that suppression of TLR3- or TLR4-stimulated IFNβ production without inhibition of IRF3 phosphorylation or nuclear translocation, or TLR7- or TLR9-stimulated IFNβ production without inhibition of IRF5 phosphorylation or nuclear translocation, could be used as a simple test to check whether a protein kinase inhibitor is likely to be a BET inhibitor.

The overproduction of IFNβ is a major cause of endotoxaemia and endotoxic shock in mice, since mice lacking expression of the genes encoding IFNβ or IFNAR are resistant to LPS-induced endotoxaemia [12,47,48]. In humans, sepsis causes 1400 deaths per day worldwide and effective therapies are still lacking [49]. It is therefore of considerable interest that the injection of I-BET into mice before the induction of septic shock with LPS or heat-killed *Salmonella enterica* serotype Typhimurium delayed or prevented the death of these mice, whereas a single injection of I-BET administered after LPS had already induced inflammation overcame this inflammatory condition [36]. BI-2536 and the closely related compound BI-6727 have passed Phase 1 clinical trials and entered Phase 2 trials for the treatment of cancer [50], and only moderate side effects of these compounds have been reported [51,52]. These compounds, as well as other BET inhibitors, therefore merit investigation as potential therapies for diseases and conditions caused by the hyperproduction of IFNβ, which include the lethal effects of flu virus as well as sepsis.

## Abbreviations

ATF2: activating transcription factor 2
BMDC: bone-marrow-derived dendritic cell
BRD4: bromodomain-containing protein 4
CBP: CREB (cAMP-response-element-binding protein)-binding protein
GAPDH: glyceraldehyde-3-phosphate dehydrogenase
HAT: histone acetyltransferase
HDAC: histone deacetylase
HSV1: herpes simplex virus 1
IFNβ: interferon β
IFNAR: Type1 interferon receptor
IRF3: interferon-regulatory factor 3
ISG: interferon-stimulated gene
JAK: Janus kinase
JNK: c-Jun N-terminal kinase
LPS: lipopolysaccharide
PLK: Polo-like kinase
pTEFb: positive transcription elongation factor B
qPCR: quantitative PCR
RANTES: regulated upon activation, normal T-cell expressed and secreted
STAT: signal transducer and activator of transcription
TBK1: TANK [TRAF (tumour-necrosis-factor-receptor-associated factor)-associated nuclear factor κB activator]-binding kinase 1
TLR: Toll-like receptor
TRIF: Toll/IL-1R (interleukin 1 receptor) domain-containing adaptor inducing IFNβ
TYK2: tyrosine kinase 2
